# Hydatid cyst in the hip joint of a young patient: a case report

**DOI:** 10.1186/s13256-026-05923-1

**Published:** 2026-03-15

**Authors:** Mohammed Ahmed Alsayyad, Hebat allah Fadhl, Mustafa Mohamed Ali Mohammed, Mohammed Saleh AlSaifi, Abdullah Ali Mohammed Al-Hutam, Mohammed Ali Saghir

**Affiliations:** 1 Faculty of Medicine and Health Sciences, Sana’a University, Sana’a, Yemen; 2 Faculty of Medicine and Health Sciences, Aden University, Aden, Yemen; 3 Faculty of Medicine, Neelain University, Khartoum, Sudan; 4 Faculty of Medicine and Health Sciences, 21 September University for Medicine and Applied Sciences, Sana’a, Yemen; 5 Modern European Hospital, Sana’a, Yemen; 6 Graduate Collage, University of Bahri, Khartoum, Sudan; 7 Cairo University Kasr Alainy Faculty of Medicine, Cairo, Egypt

**Keywords:** Hydatid cyst, Hip joint, Limping, Orthopedic, Yemen

## Abstract

**Background:**

Hydatid disease, or cystic echinococcosis, is a zoonotic infection caused by the larval stage of *Echinococcus granulosus*, predominantly affecting the liver and lungs. Musculoskeletal involvement is rare, comprising only 0.5–4% of cases, with even fewer reports of cysts in joints.

**Case presentation:**

A 12-year-old Yemeni female of Arab ethnicity was referred to a military hospital in Sanaa after presenting with septic right hip pain for over 6 months. Imaging studies revealed narrowing of the right joint space, irregularities of the femoral head and acetabulum, and slight thinning of the right femoral head articular chondral surface. Open diagnostic surgery was performed, revealing fluid-filled sacs (hydatid cysts). After 4 months of follow-up, the patient’s gait improved, and the Trendelenburg test was negative. Laboratory investigations were normal, and the X-ray showed improvements in the irregularities of the femoral head and acetabulum.

**Conclusion:**

Although uncommon, hydatid disease affecting the hip joint should be considered when diagnosing hip-related conditions, such as tuberculosis. Surgical removal and cleaning of the hip joints yield favorable functional outcomes.

## Background

Hydatid disease, also known as cystic echinococcosis, is a parasitic infection caused by the larval stage of *Echinococcus granulosus*, a small tapeworm that primarily infects dogs, which are the definitive hosts. Livestock such as sheep, goats, and cattle act as intermediate hosts, harboring the larval form of the parasite in their tissues. Humans are accidental intermediate hosts, acquiring the infection through the ingestion of eggs excreted by infected dogs. This disease remains a significant public health problem, especially in regions where close contact between humans, livestock, and dogs is common, such as parts of Africa, the Middle East, South America, and Asia (1, 2). The life cycle of *Echinococcus granulosus* involves the ingestion of eggs that hatch into oncospheres, which penetrate the intestinal wall and travel through the bloodstream to various organs. The liver is the first and most commonly affected organ, accounting for 70% of cases, followed by the lungs, which are affected in 20% of cases (3). Other organs, such as the brain, spleen, and bones, may also be involved, though these cases are rarer. The clinical presentation of hydatid disease depends on the cyst’s location, size, and the presence of complications, such as cyst rupture or infection. In many cases, cysts can grow silently for years before causing symptoms owing to their space-occupying nature, which can result in compression of surrounding tissues (4). Liver cysts may cause symptoms such as abdominal pain, jaundice, and hepatomegaly. Lung cysts, however, can present with chest pain, cough, dyspnea, and hemoptysis. If a cyst ruptures, it can lead to severe allergic reactions or anaphylaxis, which can be life-threatening (5). Secondary bacterial infection of the cyst is another potential complication, which can result in abscess formation and sepsis. The complexity of hydatid disease management is compounded by the risk of cyst rupture during treatment interventions, whether through surgery or percutaneous aspiration. Diagnosis of hydatid disease relies on a combination of clinical suspicion, imaging techniques, and serological tests. Ultrasound and computed tomography (CT) are the primary imaging modalities used to detect cysts, identify their characteristics, and assess for complications. Serological tests, such as enzyme-linked immunosorbent assay (ELISA) and indirect hemagglutination (IHA), are commonly used to detect antibodies against Echinococcus species, though their sensitivity can vary depending on the cyst’s location and integrity (4). In most cases, treatment includes surgery, percutaneous aspiration, or medical treatment with drugs that kill parasites, like albendazole or mebendazole. The choice of treatment depends on factors such as the size, number, and location of the cysts, as well as the presence of complications. The World Health Organization (WHO) has developed the PAIR (puncture, aspiration, injection, re-aspiration) technique as a minimally invasive alternative to surgery in selected cases, especially for liver cysts (6). In this case report, we present a rare case of hydatid cyst in the hip joint of a young patient.

## Case presentation

A 12-year-old Yemeni female of Arab ethnicity came to our clinic in a military hospital in Sanaa in March 2024, referred by one of my colleagues to us as a case of septic right hip. She was complaining of right hip pain for more than 6 months with limping; the pain had progressively worsened over time, limiting her daily life activities. The patient had anorexia and weight loss with no other significant medical history. Upon physical examination, the patient looked ill with no fever; the hip was tender on palpation with no skin changes over it. She had a limp on her right side; she walked on her toes owing to flexion deformity and apparent shortening.

In the supine position, she had pelvic obliquity and adduction of the right lower limb with flexion deformity of about 10–15°. She had about 25° of abduction, 10° of internal rotation, and 20°external rotation, but flexion was about 120°. There was a false leg-length discrepancy owing to pelvic obliquity and adduction of the limb. In addition, the Trendelenburg sign was positive.

Complete blood counts showed that hemoglobin (Hb) was 11.6 g/dl and white blood cell (WBC) count was 4900/mm (neutrophils 49%, lymphocytes 43%, monocytes 6%, and eosinophils 2%). Initial imaging studies, including X-rays, computed tomography (CT) scans, and magnetic resonance images (MRIs), were ordered to assess the potential bone or joint damage. In the supine position, she had pelvic obliquity and adduction of the right lower limb with flexion deformity of about 10–15°.

**Fig. 1:**
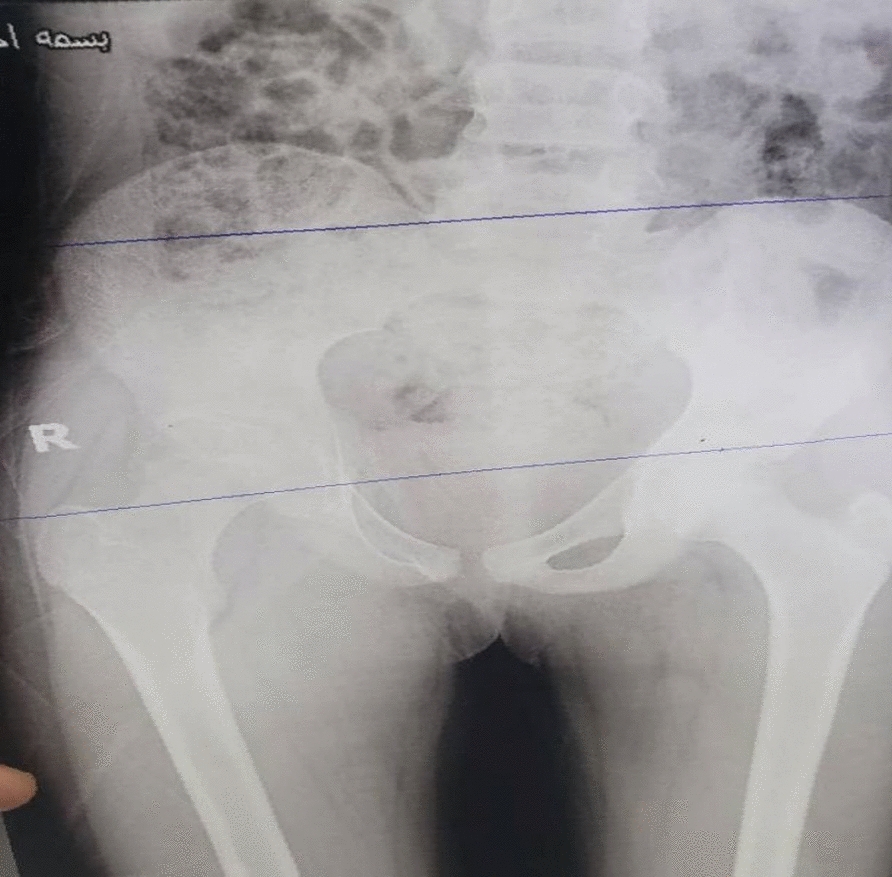
Plain radiographs anteroposterior of the pelvis and hips, which showed narrowing of right joint space with irregularities of femoral head and acetabulum

**Fig. 2:**
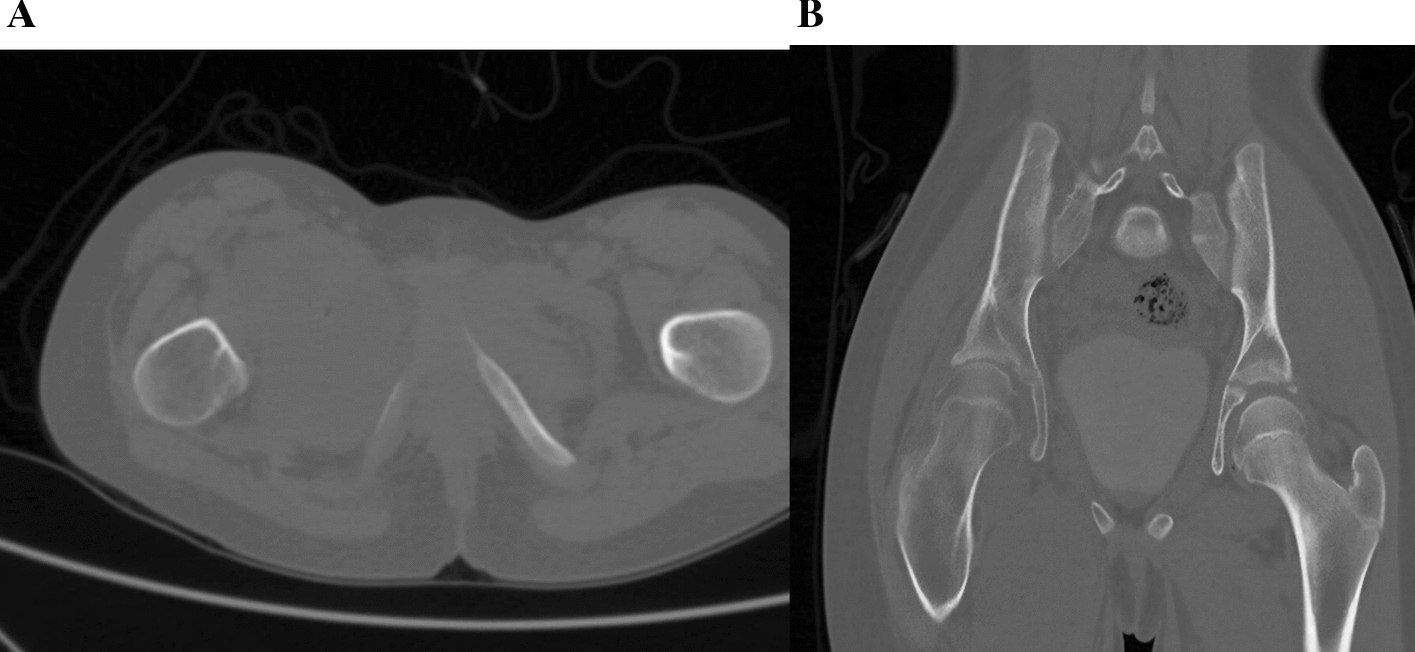
**A** Axial computed tomography image of the pelvis demonstrating a well-defined hypodense fluid collection located in the adductor muscles, suggestive of an abscess. The collection appears to displace adjacent soft tissue structures without evidence of gas formation or bony involvement. No signs of intrapelvic extension are observed. **B** Corneal computed tomography image of the pelvis bone window demonstrates significant changes in the right femoral head. There is cortical thinning and irregularity with loss of the normal spherical contour of the femoral head. Mild sclerosis is noted in the femoral neck. The adjacent acetabulum shows evidence of erosion with complete loss of the joint space

**Fig. 3:**
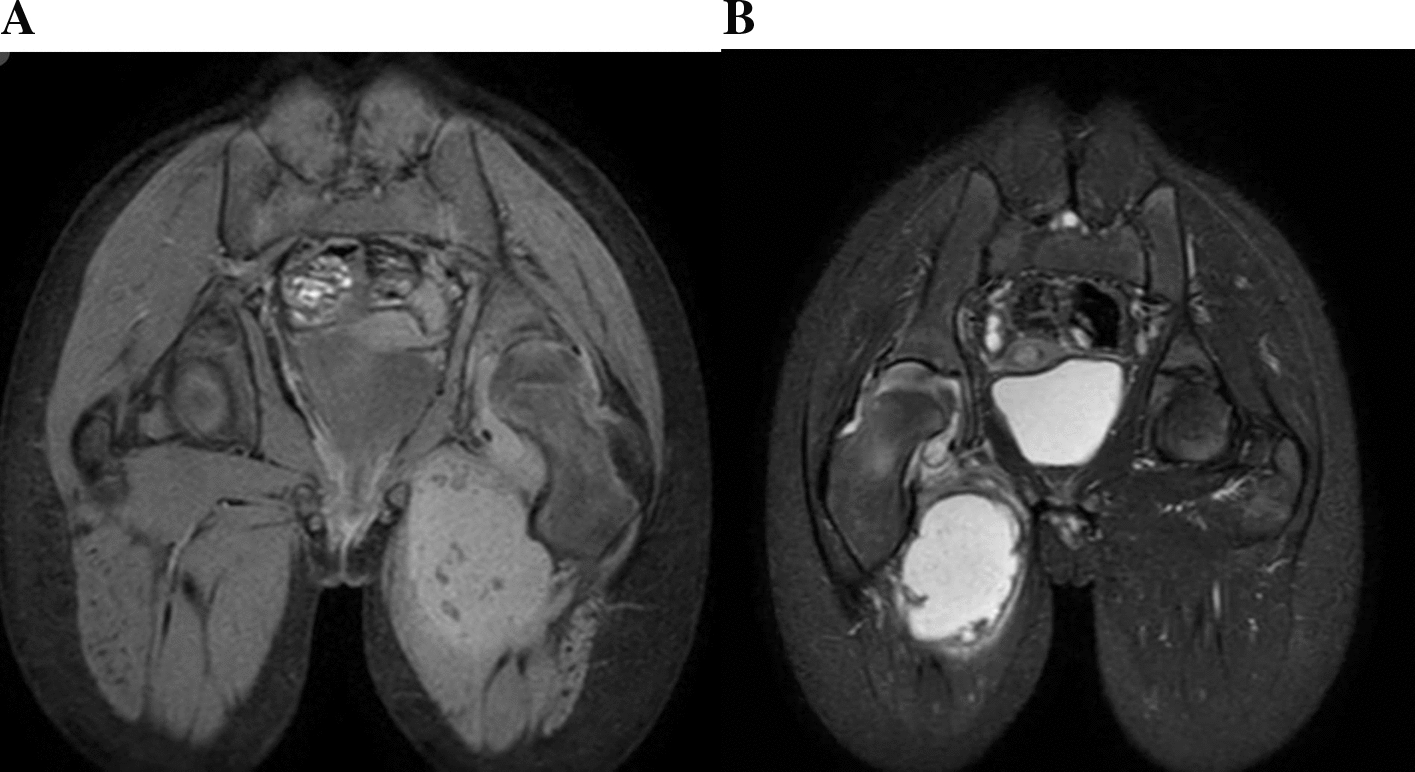
**A **and** B** Axial magnetic resonance imaging of the pelvis demonstrates a fluid collection of the fluid in the adductor region, appearing hyperintense on T2-weighted imaging and hypointense on T1, consistent with an abscess. The collection is seen communicating with the left hip joint space, raising concern for septic arthritis. Adjacent soft tissue edema is also noted, without evidence of osteomyelitis

Plain radiographs of the pelvis and hips (Fig. 1) showed a narrowing of the right joint space with irregularities in the femoral head and acetabulum. A CT scan without contrast of the hips was done; the axial CT image of the pelvis demonstrated a well-defined hypodense fluid collection located in the adductor muscles, suggestive of an abscess. The collection appeared to displace adjacent soft tissue structures without evidence of gas formation or bony involvement. There were no signs of intrapelvic extension (Fig. 2A). The corneal CT image of the pelvis bone window revealed significant changes in the right femoral head. There was cortical thinning and irregularity with loss of the normal spherical contour of the femoral head. The femoral neck exhibited mild sclerosis. The adjacent acetabulum showed evidence of erosion with complete loss of the joint space (Fig. 2B). The MRI of the pelvis showed a buildup of fluid in the adductor area, which looked bright on T2-weighted images and dark on T1, indicating an abscess. The collection’s communication with the left hip joint space raised concerns for septic arthritis. We also noted adjacent soft tissue edema, but there was no evidence of osteomyelitis (Fig. 3A and B).

The decision was open diagnostic surgery. This was conducted under general anesthesia, in aseptic conditions, with the patient positioned supine, utilizing the hip anterior approach (Smith-Petersen).

**Fig. 4:**
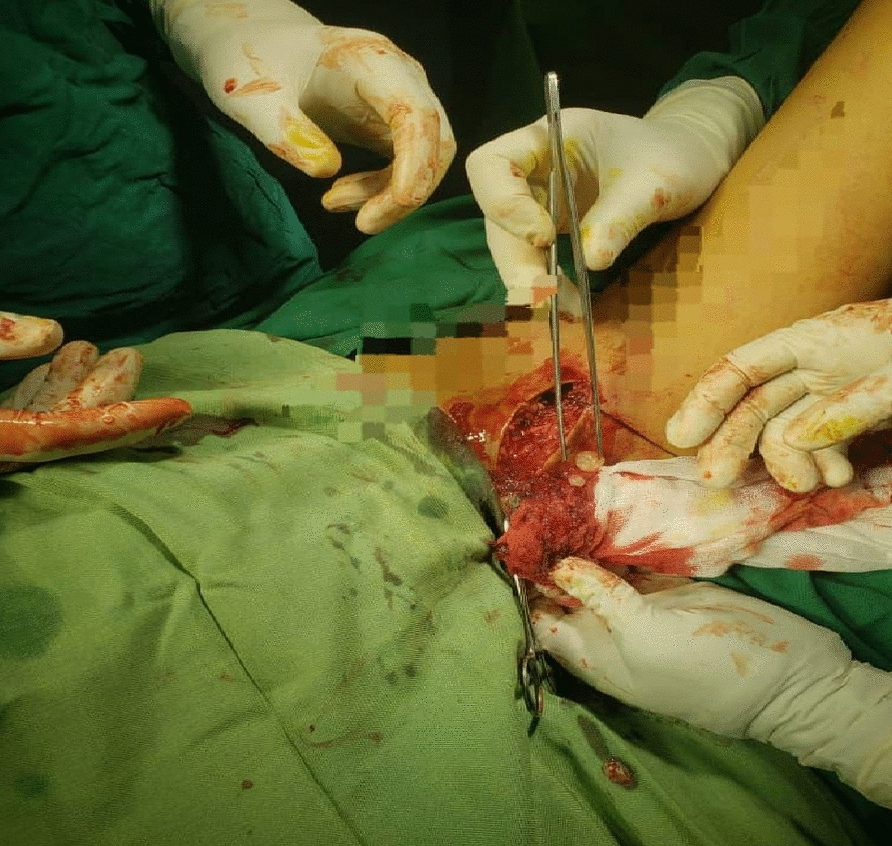
Fluid-filled sacs came out of the joint

**Fig. 5:**
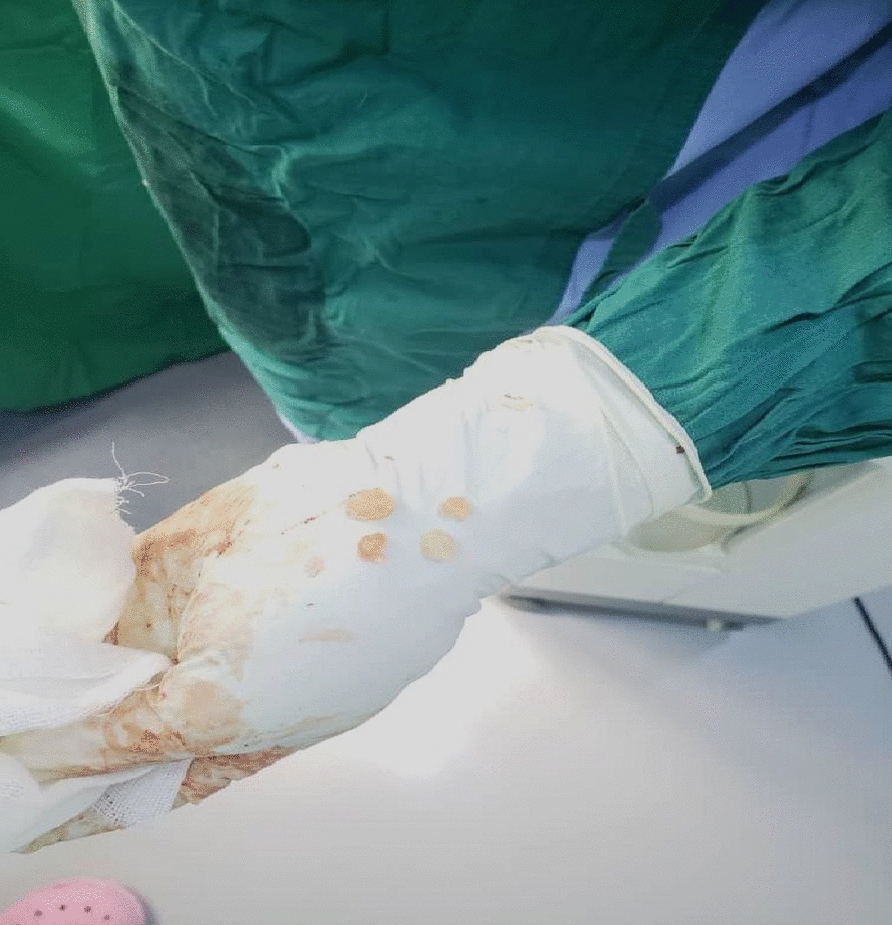
Number of hydatid cysts came out of hip joint

An incision was made from the anterior half of the iliac crest to the anterior superior iliac spine (ASIS). From the ASIS, the incision curved inferiorly in the direction of the lateral patella for 8–10 cm. Superficial dissection identified the gap between sartorius and tensor fasciae latae. Dissection proceeded through subcutaneous fat (avoiding the lateral femoral cutaneous nerve). The fascia was incised on the medial side of tensor fasciae latae. We detached the tensor fasciae latae’s origin from the iliac crest to develop the internervous plane. The ascending branch of the lateral femoral circumflex artery (crossing the gap between sartorius and tensor fasciae latae) was ligated. Deep dissection identified the plane between rectus femoris and gluteus medius. Rectus femoris was detached from both its origins. We retracted the rectus femoris and iliopsoas medially and the gluteus medius laterally to reveal the hip capsule. We adducted the hip and externally rotated it to stretch the capsule. Then, the capsule was incised; suddenly, a large number of fluid-filled sacs came out of the joint (Fig. 4). Then, after blunt dissection of the adductor muscles with flexion of the hip, with squeezing of the medial proximal part of the thigh, another number of hydatid cyst came out (Fig. 5). We collected samples for culturing, sensitivity testing, and histopathology. We irrigated the joint and the wound with a large amount of hypertonic saline. After that, we closed the wound in layers and suction drainage was done.

**Fig. 6:**
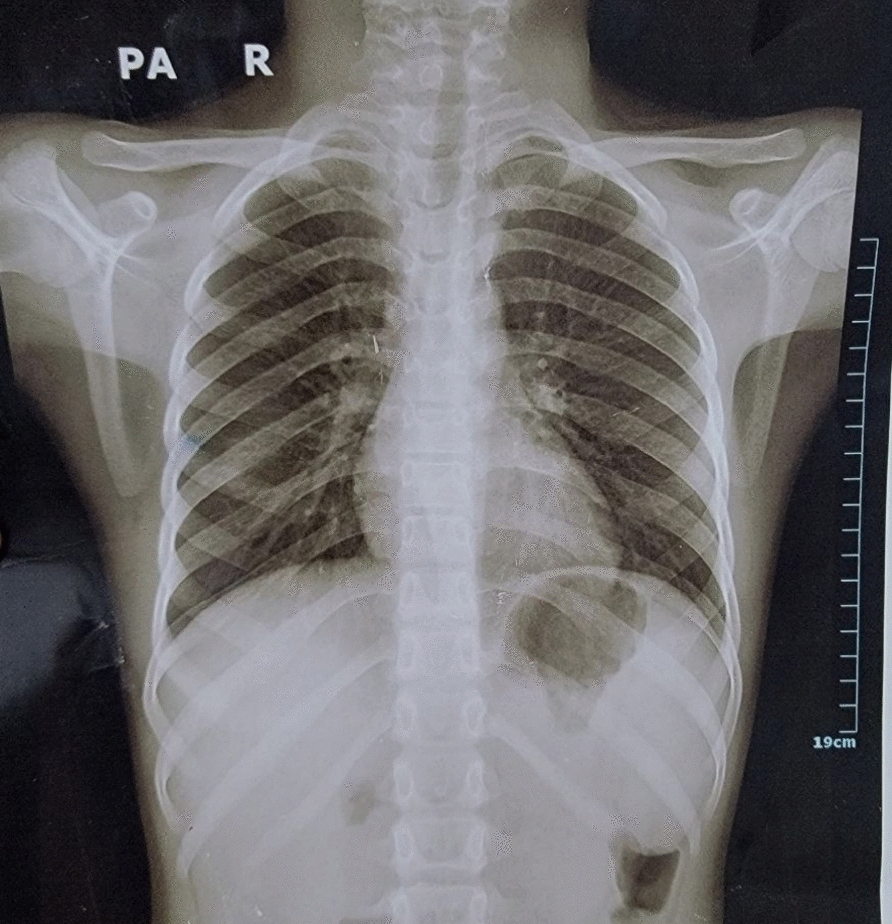
Chest X-ray was normal

Following intraoperative diagnosis of hydatid cysts, a second-day abdominal ultrasound and chest X-ray (Fig. 6) were performed to exclude involvement of the liver and chest, respectively. Both were found to be normal.

**Fig. 7:**
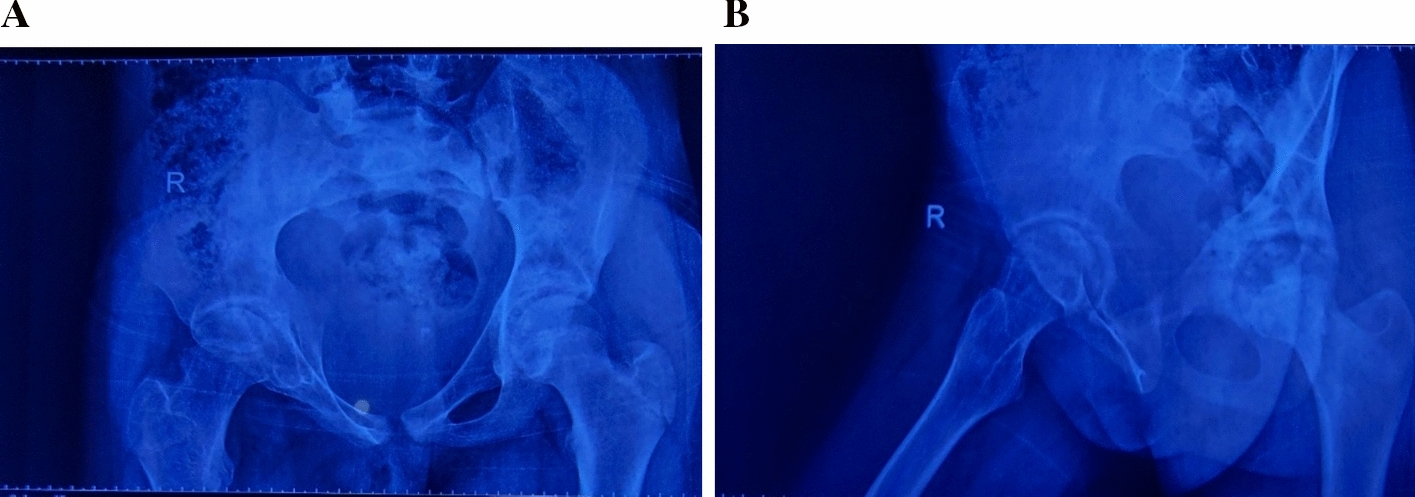
**A **and** B** Pelvic X-ray anteroposterior and laterally, respectively, 4 months postoperative showed improvement of the irregularities of the femoral head and acetabulum

Result from histopathology came after 2 weeks as cystic (hydatid cysts), and culture and sensitivity of the fluid showed no bacterial growth. After 4 months of follow-up, the case was improved. On examination, the patient’s gait was improved, as she walks normally compared with her tip-toe gait before surgery. In addition, the Trendelenburg test was negative, indicating that the abductor weakness had disappeared. Laboratory investigations were normal, and the X-ray showed improvements in the irregularities of the femoral head and acetabulum (Fig. 7A and B).

### Discussion and conclusion

*Echinococcus multilocularis*, *Echinococcus vogeli*, and *Echinococcus granulosus* are the three species that make up the genus *Echinococcus*, also referred to as tapeworm. The most common cause of hydatid disease in humans is *E. granulosus* larvae (7).

Our study presents a case of a 12-year-old female from a rural area who presented with right hip pain lasting over 6 months, accompanied by limping. Similar cases have been reported, including a 35-year-old Indian female with left hip pain and limping for a year (8), a 29-year-old Italian man who was diagnosed after fracturing his acetabulum and had a history of left hip pain for several years (9), and a 51-year-old Indian woman who presented after a pathological fracture after pain, swelling, and deformity for 2 years (10). In addition, a 35-year-old Chinese man experienced right hip pain for 5 years with progressive loss of motion (11), while a 49-year-old African woman reported pain and difficulty walking, along with a draining sinus over her left hip for 5 months (12). Furthermore, there have been cases involving patients with cemented hemiarthroplasty in Pakistan (13). In summary, these cases illustrate the complex nature of hip pain, its varying durations, and the profound impact it can have on individuals’ lives. Early diagnosis and treatment are crucial to prevent complications and improve outcomes.

Cystic echinococcosis predominantly affects the liver and lungs in humans, accounting for approximately 90% of cases. Less frequently, cysts are found in the kidneys, spleen, and muscles, with each of these locations representing about 2–3% of occurrences. Instances of cystic echinococcosis in the heart, brain, and bones are exceptionally uncommon, with each site comprising less than 1% of cases (14). Musculoskeletal involvement has been documented in approximately 0.5–4% of echinococcosis cases, primarily as a secondary implant from an initial source (15–17). The disease tends to affect areas with high blood supply. Half of the instances involve the vertebral bodies, with other common sites including the epiphysis of long bones, ileum, cranium, and ribs (16). In addition, the condition may affect the scapula, clavicle, and tarsal bones affected (18). In our situation, the clinical manifestations typically arise from complications and include discomfort, abnormal bone fractures, or joint degeneration (16). Also, we should keep in mind that differential diagnosis includes different types of tumors, such as aneurysmal bone cysts, osteoblastomas, chondrosarcoma, osteosarcoma, and metastatic lesions (19).

Ultrasonography and radiography of the chest, abdomen, and pelvis are essential for detecting visceral diseases. For accurate diagnosis, CT scans are regarded as the most effective diagnostic tool and can also be used to monitor the progression of the condition. Also, magnetic resonance imaging (MRI) is often used to find the precise location and impact on nearby structures and soft tissues, giving clear images without using ionizing radiation (20).

Treatment involves surgically removing the cysts, prescribing albendazole, and, in certain instances, performing hip replacement surgery (16). In addition to extracting the cyst surgically, prescribing albendazole (10–15 mg/kg daily) for a period of 3–6 months is necessary, with 800 mg being the highest allowable dose. An additional 3 × 600 mg of praziquantel may be included in the treatment regimen (18, 21, 22).

The case underscores the importance of considering hydatid disease in the differential diagnosis of joint abnormalities, particularly in endemic regions. A combination of imaging modalities and serological tests aided diagnosis, while treatment involved a multidisciplinary approach, emphasizing surgical and pharmacological interventions. This case report highlights an unusual presentation of a hydatid cyst in the hip joint of a young patient, a site rarely documented in literature.

## Data Availability

Yes (investigation documents and video).

## Abbreviations

AP: Anteroposterior
ASIS: Anterior superior iliac spine
CT: Computed tomography
ELISA: Enzyme-linked immunosorbent assay
IHA: Indirect hemagglutination
MRI: Magnetic resonance imaging
PAIR: Puncture, aspiration, injection, re-aspiration
Rt: Right
WHO: World Health Organization
